# Long non-coding RNAs in plants: emerging modulators of gene activity in development and stress responses

**DOI:** 10.1007/s00425-020-03480-5

**Published:** 2020-10-24

**Authors:** Li Chen, Qian-Hao Zhu, Kerstin Kaufmann

**Affiliations:** 1 Institute for Biology, Plant Cell and Molecular Biology, Humboldt-Universität zu Berlin, 10115 Berlin, Germany; 2 CSIRO Agriculture and Food, GPO Box 1700, Canberra, ACT 2601 Australia

**Keywords:** Long non-coding RNAs (LncRNAs), Arabidopsis, Rice, Stress response, Development

## Abstract

**Main conclusion:**

Long non-coding RNAs modulate gene activity in plant development and stress responses by various molecular mechanisms.

**Abstract:**

Long non-coding RNAs (lncRNAs) are transcripts larger than 200 nucleotides without protein coding potential. Computational approaches have identified numerous lncRNAs in different plant species. Research in the past decade has unveiled that plant lncRNAs participate in a wide range of biological processes, including regulation of flowering time and morphogenesis of reproductive organs, as well as abiotic and biotic stress responses. LncRNAs execute their functions by interacting with DNA, RNA and protein molecules, and by modulating the expression level of their targets through epigenetic, transcriptional, post-transcriptional or translational regulation. In this review, we summarize characteristics of plant lncRNAs, discuss recent progress on understanding of lncRNA functions, and propose an experimental framework for functional characterization.

**Electronic supplementary material:**

The online version of this article (10.1007/s00425-020-03480-5) contains supplementary material, which is available to authorized users.

## Introduction

Pervasive transcription of genomes contributes to the large number of non-coding RNAs. Long non-coding RNAs (lncRNAs) are typically defined as transcripts of more than 200 nucleotides length and without any protein coding potential (Quinn and Chang 2016; Budak et al. 2020). Since discovery of thousands of lncRNAs based on genome-wide survey, the functional relevance of lncRNAs has been debated. They have been suggested to be ‘transcriptional noise’ (Hüttenhofer et al. 2005) rather than having specific biological functions (for review, see Kung et al. 2013). It is now becoming clear that lncRNAs represent a highly heterogeneous class of molecules that can be distinguished based on their biogenesis and functions, and by their position relative to other genomic features such as protein-coding genes or transposons (Yu et al. 2019) (Table 1).

**Table 1:** Comparison of typical characteristics of mRNAs and lncRNAs

Category	mRNAs	lncRNAs
Length	Longer	Shorter
Expression specificity	More constitutive expression	Most specifically expressed
Expression level	Higher expression	Lower expression
Biogenesis	RNA pol II	RNA pol II, pol III, pol IV, pol V (plant-specific RdDM pathway)
TF binding sites	Mostly in promoters, regulatory introns, enhancers	Promoters and lncRNA gene body
Processing	5′ caps and 3′ polyA tails	Most have, some without polyA tails

Most lncRNAs are located within intergenic regions although intronic lncRNAs and natural antisense lncRNAs have been reported. Specialized groups of plant lncRNAs produced by RNA polymerase IV or V are important scaffolding components in the RNA directed DNA methylation (RdDM) pathway (Chekanova 2015). Several features of lncRNAs, including transcript length, expression level and specificity, biogenesis, post-transcriptional processing and degradation, are not only different from those of protein-coding mRNAs, but also heterogeneous among the lncRNAs. Even though large numbers of lncRNAs have been identified via next generation sequencing (NGS), microarray and comparative genomics, only a small portion of lncRNAs have been functionally characterized. LncRNAs can regulate mRNA expression via *cis* and/or *trans* mechanisms, act as signals and decoys of miRNAs or RNA binding proteins, provide specificity for target molecules such as histone modifying enzymes, and function as scaffolds stitching together large molecular machinery (Wang and Chang 2011). In terms of the layers of regulation, lncRNAs can affect target gene activity at almost all levels of regulation, including chromatin, transcriptional, post-transcriptional, translational, and post-translational levels (Fatica et al. 2014; Lucero et al. 2020). In plants, lncRNAs have been shown to participate in regulation of developmental processes, biotic and abiotic stress responses, in addition to acting as modulators of the basic cellular machinery. Comparative analysis of lncRNAs in many plant species has deepened our understanding of conservation and evolution of lncRNAs. Transposable elements contributed significantly to the origin and diversification of lncRNAs in plants (Kapusta and Feschotte 2014). Many identified and experimentally verified lncRNAs have been curated and deposited into databases, making them accessible for functional studies [see, e.g., EVLncRNAs (Zhou et al. 2018, 2019), Supplemental table S1]. In this review, we summarize the characteristics and recent findings on plant lncRNA functions, and document the strategies and experimental approaches used in identification and analysis of plant lncRNAs.

## Discovery and classification of lncRNAs

**Fig. 1:**
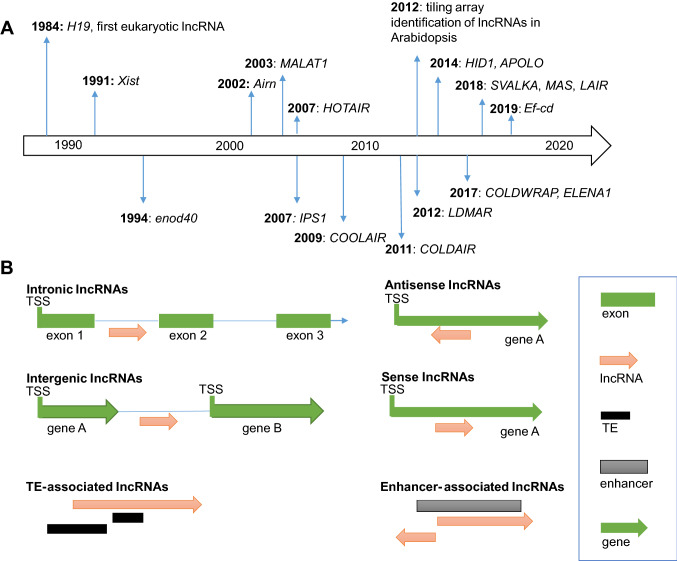
Discovery and classification of lncRNAs. **a** A timeline of lncRNA discovery. **b** Classification of lincRNAs based on genomic position [enhancer, promoter, genomic locus of protein-coding genes, transposon (TE)]

The first eukaryotic lncRNA, *H19* with a length of 2.3 kb, was discovered in mouse in 1984 and is highly expressed during embryo development (Pachnis et al. 1984). Both *H19* and its neighboring protein coding gene *Igf2* are imprinted. *H19* and *Igf2* are maternally and paternally expressed, respectively, and form the H19/IGF2 cluster (Fig. 1a) (Keniry et al. 2012; Nordin et al. 2014). Subsequently, many lncRNAs such as *Xist*, *Airn*, *MALAT1,* and *HOTAIR* were discovered and characterized in animals through genetic, molecular, and functional studies (Fatica et al. 2014). The first identified plant lncRNA, *Enod40*, was isolated as an early marker for nodule organogenesis in *Medicago* plants (Crespi et al. 1994). *Enod40* was found to trigger changes in subcellular localization of the nuclear RNA binding protein MtRBP1 (Crespi et al. 1994; Campalans et al. 2004). Since then, plant lncRNAs have been identified as regulators of miRNA activity (Franco-Zorrilla et al. 2007), epigenetic regulation (Swiezewski et al. 2009; Wu et al. 2020) and modulation of chromatin structure (Ariel et al. 2014, 2020; Kim and Sung 2018). Furthermore, the two antisense lncRNAs *LAIR* (*LRK Antisense Intergenic RNA*) and *MAS (MAF4 antisense RNA)* were found to interact with *WDR5* (a component of the COMPASS-like complex) thereby regulating flowering time in rice and Arabidopsis, respectively (Wang et al. 2018; Zhao et al. 2018).

Based on their genomic position and orientation relative to their neighboring or overlapping protein coding genes, lncRNAs can be classified into intronic lncRNAs, intergenic lncRNAs (lincRNAs), natural antisense lncRNAs, and sense lncRNAs (Ariel et al. 2015; Fig. 1b). LincRNAs can be further classified based on the genomic features with which they are associated, such as promoters, enhancers, and transposable elements (Fig. 1b).

Enhancer-associated lncRNAs (eRNAs) are usually less than 2000 nt in length and bidirectionally transcribed from corresponding enhancers, as shown in animal model systems (Shlyueva et al. 2014). These eRNAs often lack polyA tails and are degraded by the exosome when they are released from RNA polymerase II (RNA pol II, Shlyueva et al. 2014). Bidirectional transcripts are not typically detected in enhancers or promoters of Arabidopsis and other plants, most likely due to rapid degradation (Thieffry et al. 2020 and references therein). Most eRNAs are functionally uncharacterized. Data from non-plant model systems suggest roles of eRNAs in mediating changes in chromatin status, though it has also been suggested that they represent products of ‘accidental’ RNA pol II activity at enhancers (Shlyueva et al. 2014). Transposable element-associated lncRNAs (TE-lncRNAs) overlap with transposons that provide lncRNAs with distinct characteristics and chromatin environment. Transposons such as ALU elements promote nuclear localization of human lncRNAs (Lubelsky and Ulitsky 2018; Carlevaro-Fita et al. 2019). The evolutionary origins and functional diversification of lncRNAs are also influenced by transposable elements (Kapusta et al. 2013). Last but not least, many lncRNAs act as precursors of miRNAs or siRNAs, such as *Iw1* involved in the wax biogenesis of wheat (Huang et al. 2017).

Altogether, lncRNAs comprise a highly heterogeneous class of biomolecules that reflect differences in their biogenesis, functionality and turnover. In the following, we aim to provide an overview on characteristics of plant lnRNAs, pointing toward their distinct origins and mechanisms of action.

## Characteristics of lncRNAs

### Abundance and size of lncRNA transcripts

LncRNAs have been identified in a wide range of plant species including Arabidopsis, rice, and maize. The number of lncRNAs identified varies depending on the technology used for identification in each species, and large-scale analyses have reported between 6480 (Liu et al. 2012) and 6510 (Zhao et al. 2018) lncRNAs in Arabidopsis (Table 2). LncRNAs are usually shorter than protein-coding mRNAs, and they contain less exons. Some lncRNAs contain open reading frames (ORFs) with the potential of producing small peptides (Lin et al. 2020). While it is not known whether functional peptides are formed, small ORFs encoded in lncRNAs have been shown to affect growth of human cells (Chen et al. 2020).

**Table 2:** Example studies for systematic lncRNA identification in plants

Species	Tissues	Number of lncRNAs	References
*Arabidopsis thaliana*	Seedling, inflorescence	6480	Liu et al. (2012)
*Oryza sativa*	Anther, pistil, seed, shoot	2224	Zhang et al. (2014)
*Brassica rapa*	Pollen	12,051	Huang (2018)
*Gossypium hirsutum*	Root, hypocotyl, leaf, flowers	35,268	Wang et al. (2015)
*Zea mays*	Root, leaf and shoot	20,163	Li et al. (2014)
*Solanum lycopersicum*	Fruits	3679	Zhu et al. (2015)

### Expression specificity and functionality

LncRNAs are typically expressed in a more tissue-specific manner than mRNAs of protein-coding genes. In Arabidopsis, ~ 32% of lncRNAs display organ-specific expression that could be verified by experimental methods such as qRT-PCR (Liu et al. 2012). High expression specificity of lncRNAs makes them potentially suitable as markers for tissues and developmental stages. Partly, the apparent specificity could also be attributed to the generally low expression level of lncRNAs, as well as limitations in detection by standard mRNA-sequencing protocols.

### Biogenesis, splicing, and regulation of lncRNAs

As protein-coding mRNAs, biogenesis of most lncRNAs depends on RNA pol II-mediated transcription and co-transcriptional splicing. For instance, cold responsive lncRNA *SVALKA* is transcribed by RNA pol II, and it tightly regulates expression of *C-REPEAT/DRE BINDING FACTOR 1* (*CBF1*) (Kindgren et al. 2018). Additional factors or other RNA polymerases also contribute to the biogenesis of lncRNAs (Liu et al. 2015). Arabidopsis lncRNA *AtR8* is transcribed by RNA pol III and involved in the hypoxic stress response (Wu et al. 2012). A subset of lncRNAs are produced by the plant-specific RNA pol IV or pol V (Liu et al. 2015). These lncRNAs can play a role in the RdDM pathway, in which RNA pol IV-transcribed lncRNAs interact with INVOLVED IN DE NOVO 2 (IDN2), which then recruits a SWI/SNF chromatin remodelling complex to silence the activity of transposable elements (TEs) or genes by facilitating access of nucleosomes to DNA methylases (Zhu et al. 2013). Additionally, components of the miRNA pathway contribute to lncRNA biogenesis. For example, processing of a subset of lincRNAs requires *SERRATE* (*SE*), *CAP BINDING PROTEIN20* (*CBP20*), and *CAP BINDING PROTEIN80* (*CBP80*) (Liu et al. 2012). DICER-like proteins may also play roles in processing of plant lincRNAs (Ma et al. 2014). Consequently, these plant lncRNAs are usually processed into 24 nt het-siRNA by DCLs (e.g., DCL3) to methylate target genomic loci (e.g. TEs).

During RNA processing, lncRNAs are typically stabilized by capping and polyadenylation in the nucleus.  A subset of lncRNAs in mammalians, such as *MALAT1,* are processed by RNase P, do not possess polyA tails and, instead, have a specialized 3′ end structure (Wilusz et al. 2008). In humans, non-polyadenylated lncRNAs (i.e., sno-lncRNAs) that are flanked by snoRNAs and protected by RNA binding proteins have also been identified (Yin et al. 2012). Among the non-polyadenylated lncRNAs, a specialized form of RNAs called circRNAs, such as *circSEP3* in Arabidopsis (Conn et al. 2017), join their heads with tails covalently in a process called back-splicing that is mediated by the spliceosome machinery (Chen 2016). CircRNAs may regulate splicing of their cognate mRNAs, as was shown for circSEP3 and its target *SEPALLATA3* (*SEP3*) (Conn et al. 2017). Differential polyadenylation, linked with changes in preferential subcellular localization, in response to stress has been described for rice and Arabidopsis lncRNAs (Di et al. 2014; Yuan et al. 2016, 2018).

In mammalians, ~ 13% of lncRNAs are transcripts that are derived from divergent transcription in promoters of protein-coding genes (Grzechnik et al. 2014). These divergent transcripts are associated with histone modification (e.g. H3K56ac), RNA pol II Tyr1 phosphorylation and chromatin remodeling factors (e.g. SWI/SNF). Furthermore, the directionality of these divergent lncRNAs is determined by the asymmetry of U1 snRNP and polyadenylation signals (Quinn and Chang 2016). However, divergent transcription does not appear to occur in the majority of genes in *Arabidopsis thaliana* (Hetzel et al. 2016; Thieffry et al. 2020). In addition to the RNA polymerase machinery, transcription factors (TFs) and chromatin environment (e.g., histone modification and DNA methylation) also contribute to the regulation of lncRNA expression (Quinn and Chang 2016).

Data from humans suggest that splicing efficiency of lncRNAs is lower than that of mRNAs, possibly due to lower binding of splicing factors and the presence of weaker splicing-related motifs (Melé et al. 2017). Low sequencing depth and limitation of RNA-seq assembly methods may also contribute to this observation, since RACE-seq of lncRNAs detected as many alternative splicing events in lncRNAs as in mRNAs (Lagarde et al. 2016). Systematic tissue-specific interrogation of lncRNA transcripts with a higher sequencing depth and transcriptomics of specific cellular compartments, such as the nucleus, will help us to get a better overview on the lnRNA expression/abundance, the repertoire of lncRNA splice forms and other aspects of lnRNA biogenesis in plants in the future.

### Structure of lncRNAs

LncRNAs possess secondary structures which may be necessary for their functionality. There are usually two types of functional sites in lncRNAs: interacting sites which are necessary for sequence-specific interactions with RNA binding proteins, and structural sites which confer the identity of secondary and/or tertiary structures directing interacting partners (Fabbri et al. 2019). For example, *COOLAIR* participating in vernalization has a multi-way junction motif and two right-hand turn motifs (Hawkes et al. 2016), which are very conserved secondary structures in the Brassicaceae family. However, it is still unknown which proteins interact specifically with these motifs.

### Subcellular localization of lncRNAs

mRNAs are usually exported into cytosol for translation. By contrast, after processing lncRNAs can reside in nucleus or get exported to cytosol or other subcellular locations and organelles, such as mitochondria, as demonstrated by RNA FISH and ribosome profiling (Carlevaro-Fita and Johnson 2019). Data from animal model systems showed that lncRNAs are generally prone to be more enriched in the nucleus than in the cytoplasm compared to mRNAs (Derrien et al. 2012). Sequence elements within lncRNAs as well as RNA binding proteins contribute to the nuclear or cytosolic localization of lncRNAs, which reflects their cellular roles and functionality (Carlevaro-fita and Johnson 2019). For example, human lncRNAs containing ALU repeats are more prone to be retained in nucleus because of binding of specific splicing factors such as HETEROGENEOUS NUCLEAR RIBONUCLEOPROTEIN K (HNRNPK; Lubelsky and Ulitsky 2018). Some cytosolic lncRNAs are associated with mono- and poly-ribosomal complexes (see, e.g. Bazin et al. 2017; Hsu et al. 2016), and some of these lncRNAs could eventually contribute to biogenesis of small peptides. A set of nuclear lncRNAs are bound by chromatin, and this localization can be stabilized by U1 snRNP (U1 small nuclear ribonucleoprotein particle) in mammals (Yin et al. 2020). Chromatin-associated lncRNAs potentially influence TF binding or the functionality of enhancers (Shlyueva et al. 2014). While these data from animal model systems indicate intricate mechanisms underlying the subcellular distribution of lncRNAs, less is known on plant lncRNAs. Many identified lncRNAs (e.g., *COOLAIR*, *DRIR*) in plants are localized to and act in nucleus. For example, cold-induced *COOLAIR* coats the *FLC* locus in nucleus and acts in *FLC* repression by changing the histone modification status (e.g., H3K36me3) dynamics (Rosa et al. 2016; Wu et al. 2020). On the other hand, there are also cytoplasm localized cis-Natural Antisense Transcripts (cis-NATs) overlapping with protein coding genes and some of them could impact the translation of mRNAs (Deforges et al. 2019). In sum, the different types of subcellular localization point to various molecular mechanisms of action of lncRNAs in transcriptional and posttranscriptional control of gene expression.

### Decay of lncRNAs

In terms of turn-over of lncRNAs, the half-lives of lncRNAs are typically shorter than those of mRNAs, which reveals complex regulation of lncRNA metabolism in plants (Szabo et al. 2020). LncRNAs are less efficiently synthesized and rapidly degraded (Mukherjee et al. 2017). Like mRNAs, plant lncRNAs can be degraded by both 3′–5′ exonucleolysis via the nuclear exosome and 5′–3′ exonucleolysis via exonucleases such as XRN2 and XRN3 (Kurihara et al. 2012). In mutants of exosome subunits, a set of specialized lncRNAs similar to CUTs (Cryptic unstable transcripts) and PROMPTs (Promoter upstream transcripts) emerged from TSSs of mRNAs (Chekanova et al. 2007; Chekanova 2015; Thieffry et al. 2020). Data from humans suggest that exosome-regulated lncRNAs modulate the activity of enhancers, resolving deleterious R-loop structures by the exosome (Pefanis et al. 2015; Nair et al. 2020). Similar to mRNAs, the quality of plant lncRNAs is also surveilled by the nonsense-mediated mRNA decay (NMD) pathway (Kurihara et al. 2009; Kirn et al. 2009; Drechsel et al. 2013). Interestingly, the *up-frameshift* (*upf*) mutants, defective in a component of the NMD pathway, accumulate high levels of transcripts derived from antisense transcription and intergenic regions (Kurihara et al. 2009). This suggests extensive regulation of lncRNA stability via several molecular regulatory pathways.

## Functions and molecular mechanisms of lncRNAs in plants

**Fig. 2:**
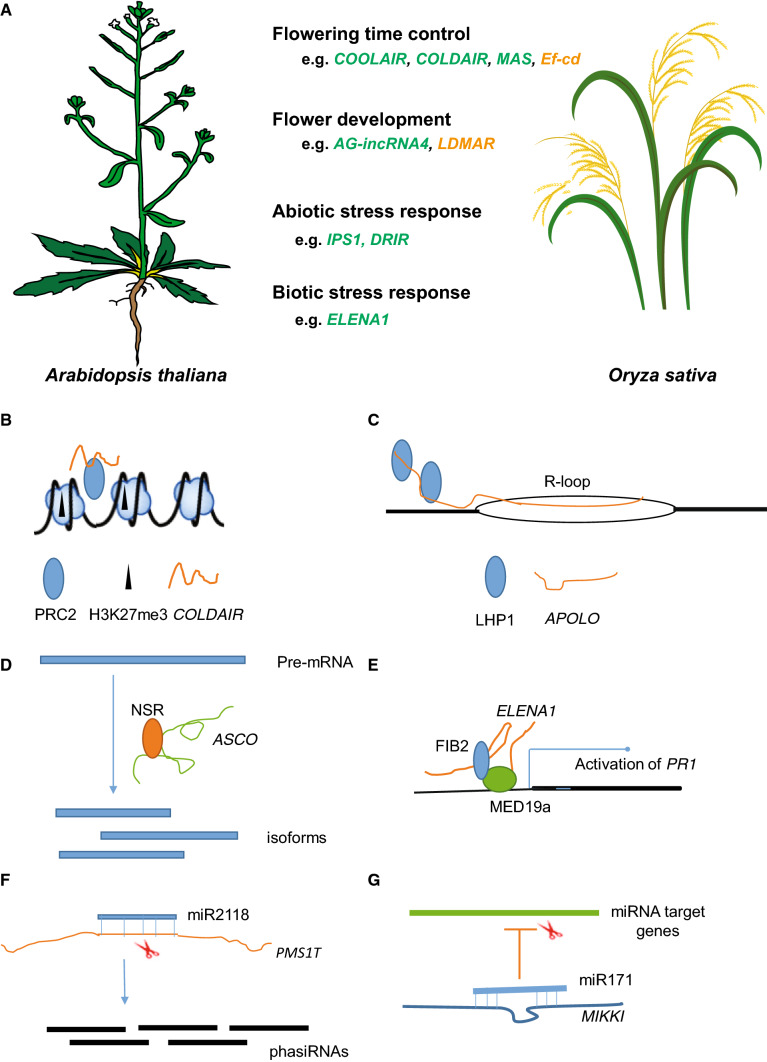
Functions of lncRNAs in plants. **a** LncRNAs participate in diverse biological processes, including flowering time control, flower development, abiotic and biotic stress responses (lncRNAs of *Arabidopsis thaliana* and *Oryza sativa* are highlighted in green and orange*,* respectively*)*. Illustrations of *Arabidopsis thaliana* and *Oryza sativa* plant are from (Illustrations 2017). **b** *COLDAIR* recruits PRC2 complex to deposit H3K27me3 marks at target gene *FLC* and thereby drives repression of *FLC*. **c** *APOLO* recognizes target gene by R-loop formation and decoys PRC1 protein. **d** *ASCO* can hijack splicing factor NSR to regulate alternative splicing of target genes. **e** *ELENA1* evicts FIB2 from the FIB2-MED19a complex and contributes to activation of *PATHOGENESIS-RELATED GENE 1* (*PR1*). **f** miR2118 targets *PM1T* to produce many phasiRNAs. **g** *MIKKI* acts as a target mimic to sequester miR171 away from its target

The recently established lncRNA database EVLncRNAs collected 1543 experimentally validated lncRNAs from 77 species, including 428 lncRNAs from 44 plant species such as Arabidopsis and rice (Zhou et al. 2018, 2019). Despite limited functional characterization of most lncRNAs, studies so far have uncovered a wide range of possible functions and molecular mechanisms mediated by plant lncRNA activities (Datta and Paul 2019) (Fig. 2a).

### Regulation of flowering time

Reproductive success in plants is tightly coupled to proper timing of the floral transition and to robust flower morphogenesis. Flowering time control in plants is regulated via internal signals such as plant hormones and environmental cues including day length and temperature. For Arabidopsis, a prolonged period of cold (winter) downregulates in a process called vernalization the expression of the major flowering repressor *FLOWERING LOCUS C* (*FLC)* to promote flowering in spring. There are several lncRNAs intricately and tightly fine-tuning the expression level of *FLC*, such as *COOLAIR*, *COLDAIR*, *ANTISENSE LONG* (*ASL),* and *COLDWRAP* (Swiezewski et al. 2009; Heo and Sung 2011; Castaings et al. 2014; Shin and Chekanova 2014; Csorba et al. 2014; Hawkes et al. 2016; Rosa et al. 2016; Kim et al. 2017; Kim and Sung 2018). *COOLAIR,* including two short and long isoforms with polyA tails, is a class of natural antisense transcripts originating from the 3′ end of the *FLC* locus (Swiezewski et al. 2009). *COOLAIR* activity is regulated by 3′ processing factors *FCA, FY, FPA, CstF64,* and *CstF77* (polyadenylation cleavage factors), and *PRP8* (the spliceosome component) (Liu et al. 2010; Marquardt et al. 2014). However, detailed molecular mechanisms of *COOLAIR* repressing *FLC* are still unknown, although the increasing level of histone demethylase FLD has been shown to contribute to H3K4me2 demethylation of *FLC* (for review, see Wu et al. 2020). *COLDAIR* is transcribed from the second *FLC* intron and acts as signal of early vernalization by recruiting the H3K27me3 writer CURLY LEAF (CLF), an enzymatic component of the PRC2 complex and a homolog of EZH2 in animals, to repress *FLC* (Fig. 2b) (Heo and Sung 2011; Kim et al. 2017). *COLDWRAP* is a lncRNA associated with the promoter of *FLC*, which also interacts with CLF to form an intragenic chromatin loop and to confer *FLC* repression (Kim and Sung 2018). Furthermore, a non-polyadenylated antisense transcript (*ASL*, for *Antisense Long*) is produced from the *FLC* locus. The function of *ASL* is still unknown but the expression level of *ASL* is downregulated in an *rrp6l* mutant (one of the exosome components, *rrp6l1 rrp6l2* double mutant) (Shin and Chekanova 2014). *MAS* (*NAT-lncRNA_2962*) is a natural antisense lncRNA from the *MADS AFFECTING FLOWERING4* (*MAF4)* locus involved in vernalization, and regulates *MAF4* via interacting with histone-modifying enzyme WDR5a (Zhao et al. 2018).

Other flowering time-related lncRNAs, including *FLOWERING LONG INTERGENIC NON CODING RNA* (*FLINC*), *CDF5 LONG NONCODING RNA* (*FLORE*), *LDMAR*, *PHOTOPERIOD-SENSITIVE GENIC MALE STERILITY 1* (*PMS1T*) and *Ef-cd*, have been recently discovered in Arabidopsis or rice (Ding et al. 2012a, b; Fan et al. 2016; Henriques et al. 2017; Severing et al. 2018; Fang et al. 2019). *FLINC* regulates ambient temperature-mediated flowering. T-DNA insertion mutants of *FLINC* flowered earlier due to upregulated *FT* expression while the underlying mechanism is not known (Severing et al. 2018). The circadian-regulated *FLORE* is a lncRNA antisense to *CDF5* and is involved in promoting of photoperiodic flowering by repression of several *CDFs* and consequently activation of *FT* (Henriques et al. 2017). In sum, the different examples indicate interesting functions for lncRNAs in the environment-dependent modulation of flowering time, providing model systems for studying how gradual changes in environmental factors trigger a defined developmental decision at the transcriptional or posttranscriptional level.

### Modulation of reproductive organ development

After floral transition, the inflorescence meristem starts to produce floral meristems, which in turn give rise to different types of floral organs. Nowadays, a number of lncRNAs such as *LINC-AP2* (Gao et al. 2016), *LONG-DAY SPECIFIC MALE-FERTILITY-ASSOCIATED RNA (LDMAR)* (Ding et al. 2012a,b), *PHOTOPERIOD-SENSITIVE GENIC MALE STERILITY T* (*PMS1T*; Fan et al. 2016), and *EARLY FLOWERING-COMPLETELY DOMINANT* (*Ef-cd*; Fang et al. 2019) have been found to regulate diverse aspects of flower and reproductive development (see Supplemental table S1 for a more comprehensive list of examples). *LINC-AP2* is an intergenic lincRNA close to the flower developmental regulatory TF gene *APETALA2* (*AP2)*. While *AP2* is downregulated upon infection with *Turnip crinkle virus* (TCV), the expression of *LINC-AP2* is elevated, and strong upregulation of *LINC-AP2* correlates with abnormal floral structures (Gao et al. 2016). The long intergenic rice lncRNA *XLOC_057324* is highly expressed in reproductive organs, and T-DNA insertion mutant analysis suggests roles in control of flowering and plant fertility (Zhang et al. 2014).

Other functions of lincRNAs include specific processes directly related to plant fertility. *BcMF11* is specifically expressed in pollen and is necessary for male fertility and pollen development in *Brassica campestris ssp*. chinensis (Song et al. 2013). *SUPPRESSOR OF FEMINIZATION* (*SUF)* is a lncRNA antisense to *MpFGMYB*, an important regulator of female sexual tissue differentiation in liverwort (*Marchantia polymorpha*). The *suf* loss of function mutant created by Cas9-based deletion displayed male-to-female sexual conversion, probably due to failure to repress *MpFGMYB* in male tissues in the absence of *SUF* (Hisanaga et al. 2019). The intronic lncRNA *AG-incRNA4* from the second intron of the floral homeotic *AGAMOUS* (*AG*) gene in Arabidopsis is expressed in leaves and interacts with the PRC2 complex component CLF to deposit H3K27me3 histone marks onto the *AG* locus, thereby contributing to repression of *AG* expression in leaves. Knockdown of *AG-lincRNA4* resulted in *AG* activation in leaves by lowering the H3K27me3 level at the *AG* locus. Consequently, the corresponding mutant showed phenotypes resembling those of ectopic *AG* expression (Wu et al. 2018). *LDMAR* was identified in rice through map-based cloning and regulates photoperiod-sensitive male fertility via RdDM (Ding et al. 2012a, b; Zhou et al. 2012).

Small RNAs, including het-siRNAs, phase-siRNAs, and miRNAs, play a critical role in development and stress responses. For example, miR396-mediated regulation of *HaWRKY6* plays a role in protection of damage caused by high temperature in sunflower and affects plant growth (Giacomelli et al. 2012). Identification of ncRNA-W6 (*ncW6*) in the promoter of *HaWRKY6* revealed another layer of regulation of the gene by a non-coding RNA. *ncW6* derives from a transposon of the MITE family and is able to form a hairpin structure that is processed into 24 nt het-siRNAs by DCL3 to trigger DNA methylation in the flanking regions of *HaWRKY6*. DNA methylation changes chromatin structure of the *HaWRKY6* locus and promotes the formation of a loop encompassing the whole locus to enhance transcription of *HaWRKY6*. The level of DNA methylation, and consequently, the formation of the loop and the expression level of *HaWRKY6 are* regulated in a tissue-specific manner (Gagliardi et al. 2019)*.* Another lncRNA, *PMS1T*, identified by map-based cloning in rice, contributes to photoperiod-sensitive male sterility by producing phase-siRNAs in a miR2118-dependent manner (Fan et al. 2016) (Fig. 2f). *Ef-cd* is an antisense RNA in the *OsSOC1* locus and positively regulates *OsSOC1* activity by deposition of H3K36me3, thereby reducing the time-span that is needed to reach plant maturity without yield penalty (Fang et al. 2019). Together, these findings highlight important functions for lncRNAs in reproductive growth via different molecular mechanisms. Since many uncharacterized lncRNAs are associated with genomic loci that encode developmental control genes, these will provide interesting targets of future research.

### Response to abiotic and biotic stresses

As sessile organisms, plants must cope with various kinds of abiotic and biotic challenges. Plants have evolved intricate signaling cascades and molecular networks to combat these stresses. Under phosphate starvation conditions, Arabidopsis plants express the lncRNA *Induced by Phosphate Starvation 1* (*IPS1*). *IPS1* acts as an endogenous target mimic to sequester and repress miR399, a repressor of *PHOSPHATE2* (*PHO2)*, which encodes a ubiquitin-conjugating E2 enzyme. Repression of *PHO2* enhances phosphate uptake and accumulation (Fig. 2g) (Franco-Zorrilla et al. 2007). *ELF18-INDUCED LONG-NONCODING RNA1* (*ELENA1*) is a 589-nt lincRNA conferring immunity of Arabidopsis. Plants with a reduced expression level of *ELENA1* by an artificial miRNA are more sensitive to the bacterial pathogen *Pseudomonas syringae pv. tomato* DC3000 and show downregulation of several immunity marker genes, including *PATHOGENESIS-RELATED GENE 1* (*PR1*). In contrast, overexpression of *ELENA1* activates immune genes such as *PR1*. *ELENA1* exerts its role via interacting with components of Mediator (Fig. 2e) (Seo et al. 2017). The lncRNA *DROUGHT-INDUCED LNCRNA* (*DRIR)* in Arabidopsis positively regulates salt and drought response. Plants overexpressing *DRIR* showed enhanced salt and drought tolerance and displayed higher survival rates under salt and drought stress conditions (Qin et al. 2017). Many other stress response-related lncRNAs have been identified, but their molecular mechanisms of action are yet to be investigated (see, e.g. Zhu et al. 2014; Wang et al. 2017).

### Functions in other biological processes

LncRNAs have been shown to participate in diverse biological processes, such as leaf development, auxin signaling, and photomorphogenesis. *TWISTED LEAF* (*TL*) is a rice lncRNA antisense to *OsMYB60* and required for maintaining leaf blade flattening by regulating the expression of its sense mRNA (Liu et al. 2018). The auxin responsive Arabidopsis lncRNA *APOLO* plays a role in fine-tuning the transcription of its neighboring *PINOID* (*PID*) gene, an important regulator of auxin polar transport, via formation of a chromatin loop involving the promoter of *PID*. The expression level of *APOLO* determines the chromatin environment in the promoter region of *PID* affecting histone modifications and the level of DNA methylation, and consequently the formation of the chromatin loop and the expression level of *PID* (Fig. 2c) (Ariel et al. 2014). In addition to these *cis* effects, *APOLO* also regulates target loci in *trans* by formation of R-loop (DNA-RNA duplexes) mediated by short sequence complementarity and thereby decoying PRC1 to target loci to modulate their chromatin status (Ariel et al. 2020). Furthermore, the photomorphogenesis-related lncRNA *HID1* (*HIDDEN TREASURE1*) represses the transcriptional activity of its target gene *PHYTOCHROME INTERACTING FACTOR 3* (*PIF3).* HID1 forms a large nuclear complex with as yet unknown proteins and modulates the chromatin structure in the *PIF3* promoter, consequently repressing hypocotyl elongation of Arabidopsis seedlings (Wang et al. 2014).

LncRNAs function in basic nuclear regulatory processes by interacting with proteins. For example, nuclear speckles are nuclear domains enriched with splicing-related factors and located in interchromatin regions of nucleoplasm (Spector and Lamond 2011). It was shown that Arabidopsis *ASCO-lncRNA* competes for the NUCLEAR SPECKLE RNA-binding proteins (NSRs) and sequesters NSRs to modulate the alternative splicing pattern of target genes (Fig. 2d) (Bardou et al. 2014). LncRNAs are also components of the telomerase molecular machinery. For example, lncRNA *AtTR* is the RNA subunit of telomerase, which interacts with *TELOMERASE REVERSE TRANSCRIPTASE* (*TERT*) to maintain the integrity and stability of telomeres (Michal et al. 2019; Song et al. 2019). This indicates roles of lncRNAs in genome integrity and genome functions beyond biological functions in development or environmental response, which emphasize the need for multiscale experimental methodologies to characterize lncRNA functions.

## Experimental methodologies for functional characterization of lncRNAs

Similar to  protein-coding genes, functions of lncRNAs can be investigated using forward and reverse genetics approaches. However, functional analysis of lncRNAs is hampered by the need to distinguish functions of the lncRNA transcript from that of its genomic locus. This is because lncRNAs are often produced from DNA genomic regions with other functions, e.g., loci of protein coding genes (in the case of intronic or antisense lncRNAs) or enhancers (e.g., in the case of eRNAs). Also RNAi-based knockdown of lincRNA activities can have side effects that are not related to the functions of lincRNAs, for instance, RNAi-mediated DNA methylation is possible to change the functionality of the genomic regions in other aspects (e.g., affecting enhancer activity). Finally, not the lincRNA transcript itself, but the process of transcription may exert a regulatory function (Gowthaman et al. 2020).

In plants, a small set of lncRNAs has been identified by map-based cloning and functionally characterized, such as *LDMAR* (Ding et al. 2012a), *PMS1T* (Fan et al. 2016), *Ef-cd* (Fang et al. 2019) and *Iw1* (Huang et al. 2017). However, reverse genetics (e.g., based on T-DNA mutagenesis populations, RNAi, overexpression) is most commonly used for studies of lncRNA functions, because the vast majority of lncRNAs were identified by high throughput technologies. Every method used to perturb lncRNA functions has disadvantages. For example, T-DNA insertions or CRISPR/Cas9-based deletions in intergenic regions may not only inhibit lncRNA expression, but also affect other functions of the DNA sequences, such as TF binding sites or regulatory elements within lincRNA loci, thereby altering the expression of nearby protein coding genes. When studying antisense, sense, or intronic lncRNAs, these approaches can also have side effects, such as modifying splicing of the associated protein-coding genes. The RNAi technology on the other hand is known to be prone to off-targeting, and may cause RdDM, thereby confounding functional interpretation of the target lncRNAs. Thus, a combination of different approaches and proper control experiments are required to study lncRNA functions.

**Fig. 3:**
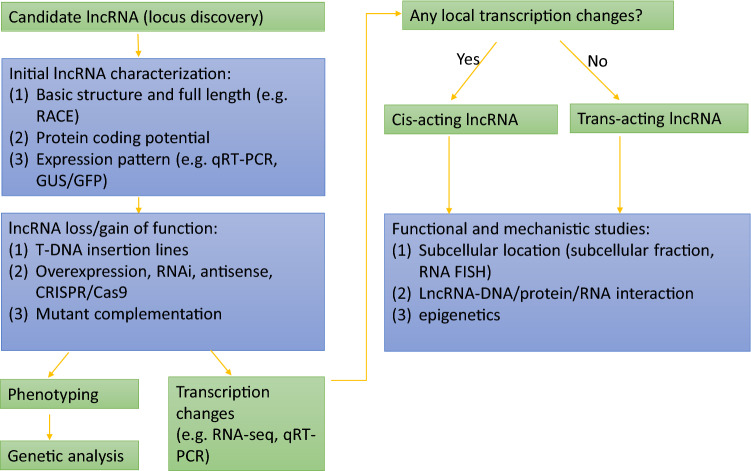
Experimental workflow for dissection of lncRNA functions. Details are described in the main text

Here, we propose a workflow for functional investigation of plant lncRNAs (Fig. 3). When a candidate lncRNA is identified, the first task to perform a comprehensive inspection of the sequence and structure of the lncRNA. Rapid amplification of cDNA ends (RACE) can be used to obtain the full length transcript(s) of the lncRNA. Searching publicly available datasets, such as cap analysis of gene expression (CAGE) and polyA site sequencing (PAS-seq) (Shepard et al. 2011), and performing RNA-seq will give clues about the general structure as well as alternative splicing patterns of the lncRNA locus of interest. Northern blotting and quantitative RT-PCR (qRT-PCR) are standard approaches for investigation of the expression profiles of lncRNAs. GREEN FLUORESCENT PROTEIN (GFP) reporter imaging can be used to study dynamic lncRNA promoter activity. RNA-FISH allows study of the activity and localization of lncRNAs to the level of individual genomic loci (Rosa et al. 2016). Recent studies showed that some lncRNAs could translate into small peptides, and thus it is necessary to distinguish whether the lncRNA of interest functions as non-coding RNA or as small peptide. Several bioinformatics and experimental approaches can be employed for this purpose, such as CPC2 to test for coding potential test (Kang et al. 2017). Additionally, lncRNAs should be queried in protein databases including Pfam (Finn et al. 2016) and Uniprot (The UniProt Consortium 2017) to know whether they have potential homologous proteins. Ribosome footprints based on ribosome profiling are indicative of open reading frames, which are used to discriminate lncRNAs from protein coding genes (Lander 2014; Hsu et al. 2016; Bazin et al. 2017). Loss/gain-of-function mutants are generated to investigate functionality of the lncRNA. Since every technique has its own limitations (see above), it is necessary to use multiple different approaches such as T-DNA mutagenesis, RNAi, overexpression with constitutive and tissue-specific promoters, and CRISPR/Cas9-based mutagenesis combined with mutant complementation. A large number of publicly available T-DNA insertion lines are available for both Arabidopsis and rice. Analysis of independent mutant alleles and, importantly, transgenic mutant complementation (in *trans*) can be used to validate the functionality of lncRNAs (see, e.g. Fang et al. 2019). When a lncRNA has multiple isoforms, generating mutants for each isoform can distinguish the roles of individual isoforms. CRISPR/Cas9-based mutagenesis usually creates small indels in the target site (Li et al. 2018), which might not influence the functionality of the lncRNA. This can be overcome by introducing a pair of single guide RNA (sgRNA) to induce a larger indel in the corresponding lncRNA locus. Use of multiple such pairs of sgRNAs covering the entire lncRNA can help to dissect functional regulatory sites of the lncRNA. In these experiments, potential side effects arise from mutagenizing other functional DNA elements that reside within the lncRNA locus. Therefore, the target lncRNA locus should be evaluated carefully by taking into account existing information on TF binding sites or chromatin structure. In all types of mutant analyses, the phenotypic analyses should be complemented by monitoring changes in expression of the protein-coding genes flanking the lncRNA locus of interest. Especially for studying *trans* mechanisms of lncRNAs, (inducible) ectopic expression or artificial miRNA technology can be used for validation.

Functional lncRNAs typically interact with DNA, RNA, and proteins. The in vivo or in vitro approaches developed for investigating the RNA–protein (e.g., RIP and CLIP) (Cao et al. 2019), RNA-DNA (e.g., ChIRP) (Chu et al. 2012), and RNA-RNA (e.g., RAP-RNA)(Engreitz et al. 2014) interactions can be used to identify the molecular partner(s) interacting with lncRNAs. The subcellular localization of lncRNAs is also important, since it may provide clues on functions. For example, single molecule RNA FISH analysis revealed that *COOLAIR* and *FLC* transcripts are mutually exclusively expressed (Rosa et al. 2016). It is important to further develop in vitro and in vivo experimental methods to screen and validate the interaction between lncRNAs and their partner molecules. For example, a trimolecular fluorescence complementation (TriFC) system has been used to demonstrate lncRNA-protein interaction by tagging a lncRNA with the MS2 system (MS2 sequence and phage MS2 coat protein fused to YFP-N) and co-transfecting it together with the YFP-C tagged RNA-binding protein into tobacco leaves  via *Agrobacterium* (Seo et al. 2019). Finally, we envision that efficient novel experimental and computational methods will be developed for investigation of the functionality of lncRNAs in plants at the level of single cells or subcellular compartments.

## Conclusions and perspectives

Mounting evidence shows involvement of lncRNAs in wide ranges of biological processes, including development and stress responses. Efficient computational methods are urgently needed to predict functional lncRNAs for experimental validation. LncRNAs act in *cis* or in *trans* to regulate the function of their target genes through diverse mechanisms that involve interactions with DNA, RNA or proteins. Many plant lncRNAs (e.g., *COLDAIR*) function epigenetically to modulate the expression of their target genes by modifying histone modification status and chromatin organizations. However, despite the diversity of molecular mechanisms and functions, our understanding of most plant lncRNAs is still elusive and unclear. There are at least a couple of reasons. Firstly, the effects of lncRNAs might only be observed under specific conditions given that expression of most lncRNAs is highly tissue/condition-specific. Secondly, lncRNAs represent a heterogeneous group of RNA molecules in plants. Several subclasses of lncRNAs (e.g., enhancer RNAs) are largely coupled with regulatory DNA sequences (e.g., TFBSs), which makes it difficult to assess their (if any) specific functions. Development of technologies is imperative to understand molecular mechanisms of lncRNAs (Ariel et al. 2020). Large-scale functional screens of lncRNAs by CRISPR/Cas9-based mutagenesis have been established in human and flies, although only a small percentage of lncRNAs showed context-specific phenotypic changes (Liu et al. 2017). A similar system has yet to be developed for plant lncRNAs although large-scale mutagenesis populations have been created in several plant species by transformation of sgRNA libraries targeting protein-coding genes (Jacobs et al. 2017; Lu et al. 2017; Meng et al. 2017; Zhang et al. 2019; Liu et al. 2020; Bai et al. 2020). Finally, we need to investigate how we can effectively utilize the knowledge on beneficial lncRNAs in breeding programs to develop novel plant germplasm and elite crop varieties. An excellent example for this is provided by *Ef-cd* that promotes early maturity without yield penalty probably due to better nitrogen utilization and photosynthesis in rice. It functions like a dominant gene as plants homozygous or heterozygous for *Ef-cd* showed better agronomic performance compared to plants without *Ef-cd*. It thus is valuable for rice breeding. Fang et al. (2019) have developed molecular markers completely linked with *Ef-cd*, which can be used to identify new early maturity rice germplasm containing *Ef-cd* and to introgress *Ef-cd* into elite rice cultivars to further improve their maturity and agronomic performance based on marker-assisted selection. For *LDMAR* and *PMS1T*, base editing can be used to change the unfavorable alleles into favorable ones as single nucleotide polymorphisms seem to be the cause for changes in fertility. These examples show that utilizing knowledge on plant lncRNA functions can open new possibilities for plant breeding research, thereby improving crop quality and performance.

### *Author contribution statement*

LC conceived the topic of the article. LC, KK, QHZ contributed to the writing of the manuscript. All authors read and approved the final version of the manuscript.

## Electronic supplementary material

Below is the link to the electronic supplementary material.

Supplementary file1 (XLSX 15 kb)
